# The Development of an Accreditation Framework for Continuing Education Activities for Pharmacists

**DOI:** 10.3390/pharmacy8020075

**Published:** 2020-04-28

**Authors:** Sarah Drumm, Frank Moriarty, Michael J. Rouse, David Croke, Catriona Bradley

**Affiliations:** 1 Irish Institute of Pharmacy, Royal College of Surgeons in Ireland, D02 FP84 Dublin, Ireland; catrionabradley@rcsi.com; 2 HRB Centre for Primary Care Research, Department of General Practice, Royal College of Surgeons in Ireland, D02 YN77 Dublin, Ireland; frankmoriarty@rcsi.ie; 3 International Services Program, Accreditation Council for Pharmacy Education, Chicago, IL 60603, USA; mrouse@acpe-accredit.org; 4 Quality Enhancement Office, Royal College of Surgeons in Ireland, D02 YN77 Dublin, Ireland; dtcroke@rcsi.ie

**Keywords:** accreditation, framework, pharmacy, continuing education, consensus

## Abstract

Accreditation is the recognition that an educational activity meets certain standards. The processes for accreditation vary considerably depending on the type of activity, and currently there are differing accreditation systems in place for pharmacy continuing education (CE) across different countries. Research was carried out on a selection of these systems with the aim of developing a catalogue of accreditation approaches, and exploring the possibility of developing a common framework for the accreditation of pharmacy CE activities. Accreditation processes from the countries represented by the Global Forum on Quality Assurance of Continuing Education and Continuing Professional Development (GFQACE) were reviewed to explore the themes and patterns in them. This informed the development of a proposed accreditation framework for CE activities for pharmacists. A Delphi method over four rounds involving seven participants from each GFQACE organisation was used as a consensus building technique. Agreement was achieved on including 15 items in the framework within four stages (Input, Process, Output, and Quality Improvement). The GFQACE steering group indicated their intention to use the resultant framework as the basis for the exploration of mutual recognition of accreditation between member countries.

## 1. Introduction

Following the completion of formal education, the maintenance of knowledge and the importance of continuing to engage in education has been recognised across many professions [1,2,3,4,5,6]. In 1900, Sir William Osler, one of the founders of the Johns Hopkins University School of Medicine, gave a lecture in London on “The Importance of Post-Graduate Study” and proclaimed that “If the licence to practise meant the completion of his education how sad it would be for the doctor, how distressing to his patients!” [7]. The mechanism for continuing education in the past was through attendance at lectures, reading journals, etc., activities that were largely carried out on an ad hoc basis. One of the challenges of this was to ensure that the activities were of a sufficiently high quality, and that there was consistency across different organisations, which is where accreditation fits into the picture.

Accreditation is a process of “review and approval” [8], and the value of accreditation is “entirely dependent on rigorous application of appropriate principles and rules” [9] (p. 2). Accreditation is therefore an indicator of formal recognition that an organisation or an activity meets certain criteria, with the aim that this provides a measure of quality assurance [10,11,12,13]. It provides both a threshold for quality and a mechanism for improving quality [14]. Accreditation can be described as a “trust-based, standards-based, evidence-based, judgment-based, peer-based process” [15] (p. 5). This description incorporates both the scientific area of evidence, along with the opinion of peers and experts. Accreditation is used in different industries and environments including the education, software, and food sectors, and can be used for developing new standards or upgrading existing standards, with continuous improvement as a core feature [16]. In educational environments, the independence of accreditors from third-party influence is vital to ensure that the learning activities or organisations are free from commercial interests [9].

But what does an accreditation framework look like, and what should it include? To answer these questions, the approaches used by organisations engaged in accreditation for pharmacy continuing education were explored. Opinions were sought on what constitutes a robust accreditation framework that would assure quality in the provision of continuing education for pharmacists. 

The organisations that participated in this research were those of the Global Forum on Quality Assurance of CE/CPD (GFQACE). This is a group comprising representatives from organisations with a remit for national regulation and/or management of quality assurance of CE activities for pharmacists. The aim of the group was to provide an informal forum in which attendees with similar remits could network and share insights and ideas relating to the quality assurance of continuing education in pharmacy. The participating organisations were:

- Accreditation Council for Pharmacy Education, United States (ACPE)
- Australian Pharmacy Council (APC)
- Canadian Council on Continuing Education in Pharmacy (CCCEP)
- Irish Institute of Pharmacy (IIOP)
- Pharmaceutical Society of New Zealand (PSNZ)
- Royal Pharmaceutical Society, United Kingdom (RPS)
- South African Pharmacy Council (SAPC)

It was proposed within the GFQACE that the development of a framework for accreditation of pharmacy continuing education would be beneficial in light of evolving requirements for continuing professional development internationally. Such an initiative could facilitate future projects of mutual recognition of accreditation and could be shared with other organisations with similar interests as a stepping stone towards the development of a global framework. The research presented in this paper was undertaken to inform the work of the GFQACE. 

## 2. Methods 

The study was conducted between November 2017 and February 2019, and involved a cross-sectional questionnaire, followed by a Delphi study. Further details are provided on these processes in this section. The initial phase of the research was to review the accreditation processes from the countries represented by the GFQACE and to identify key themes and patterns. A convenience sample was used, which consisted of representatives of all the bodies participating in the GFQACE (n = 7). Each participant was selected by their organisation as the staff member most qualified to respond, as all had relevant expertise, experience, and interest in the area of accreditation. 

An online questionnaire was used to gather information about existing accreditation frameworks from the participating organisations. The questionnaire consisted of 23 questions, and took approximately 30 minutes to complete. It was developed to cover the various aspects of accreditation, from the initial identification of whether an activity or training provider is eligible to submit an application, through to the application process and the outcome at the completion of the process. The objective of collecting the data from the questionnaire was to compare accreditation processes, look for patterns, and determine areas of similarity and difference across the organisations. To ensure the face validity of the questionnaire, the questions were reviewed, and the functionality of the questionnaire was pilot tested by four members of the research team. 

The questions in the questionnaire were mostly open, with free text boxes. Additionally, participants were given the opportunity to submit documents relating to the accreditation process. Guidance was given on the type of documentation required including policy or guidance documents on accreditation, legislative guidance where relevant, an accreditation application form, and/or any paperwork an applicant must submit relating to accreditation, links to the organisation website where data and documents can be found, and any other relevant information.

The format of the questionnaire moved from the general questions to those that were more specific, following the idea of the ‘funnel’ design where the questions progress from the general to the particular with questions grouped together by topic [17,18]. The questionnaire broadly followed the accreditation journey beginning with the accrediting body itself, moving through the steps in the process to the conclusion where the outputs from the process can be seen. There were four sections in the questionnaire, each addressing a distinct area, as can be seen in Table 1. 

A free text box was provided at the end of the questionnaire for any additional comments the respondent might have. The collection procedure used for the questionnaire was not anonymous, as further information may have been required from respondents following completion of the questionnaire. The full questionnaire can be viewed in Appendix A. 

For this study, as the responses were reviewed on the basis of frequency of occurrence in addition to the content, this makes use of both quantitative and qualitative analysis. Using both methods provides a more comprehensive understanding of the data, looking at both the prevalence of occurrence and the how of an item, while hopefully taking advantage of the strengths of each of the approaches and reducing any bias or weaknesses caused by the use of a single methodology [19,20,21,22,23,24,25]. Documents were requested from participants to provide a fuller picture of the accreditation process, and so that the contents of the application form and standards could be reviewed. The documents provided were reviewed to look for commonalities and differences amongst them, and to fill in any gaps in the information provided by the questionnaire. Without this extra documentation, it would not have been possible to fully explore the commonalities across the organisations. The questionnaire looked to collect information, that is to say that what was being collected was the content of the responses. As the content of the responses was reviewed, key themes and patterns were identified and used to develop the framework for the next stage of the study. An inductive approach was used as the framework was only developed subsequent to the completion of the questionnaire.

The next phase was to develop a proposal for an accreditation framework. This was developed informed by the following: the responses to the questionnaire; the supporting documentation provided by the GFQACE; a review of the literature on accreditation, continuing education, continuing professional development and quality; and the model for Pillars and Foundations of Educational Quality developed with the International Pharmaceutical Federation (FIP) [26,27,28]. The aim of the FIP model is to provide a structure for educational quality and describe the “key principles and elements that should be included in the accreditation process” [28] (p. 13) and it includes the pillars of context, structure, process, outcomes, and impact. By using the themes and patterns identified from the questionnaire and the supporting documentation, along with the FIP model, a framework was developed, which was hoped to be reflective of the accreditation processes in other organisations and would be aligned with best practice. 

The method used to validate the proposed framework was a consensus building technique using the Delphi method, where the framework was circulated to the GFQACE. Table 2 shows the accreditation framework as circulated to the participants in round one of the Delphi. This shows each stage (e.g., input) and the subsections in the stage (e.g., context for activity). A description of what this constituted, why it was required, and who provides it (i.e., the applicant or the accrediting body), was detailed in the framework. 

The initial aim was to complete three rounds of the Delphi, circulating feedback after each round, and this process was granted ethics approval by the Royal College of Surgeons in Ireland (RCSI) Research Ethics Committee. The participants in the Delphi were representatives from the GFQACE, with one person from each of the seven organisations responsible for completion of the Delphi. Similar to the questionnaire, and with the same rationale for employing it, an online form was used for the Delphi and prospective respondents were provided with a link to the form. The basic structure of the Delphi involved asking the respondent to rate their agreement or otherwise to a statement using a 5-point Likert Scale (the 5-point scale used in this study will hereafter be referred to as “the scale”), which ranged from “strongly disagree” (1) to “strongly agree” (5). Additionally, there was a free text box for comments. Respondents were asked to complete the comment section, indicating why they chose that rating on the scale as well as any suggested modifications. The comment section was mandatory, but if they had nothing to add, they were advised that they could enter NA into the box.

The following were the a priori cut-off scores for determining whether items would be carried forward to the next round or eliminated:

- No items to be eliminated based on the scores from round one.
- At the end of round two, items rated as disagree or strongly disagree by at least five of the seven respondents will be excluded. All other items will be carried over to round three.
- At the end of round three, items rated as agree or strongly agree by at least five of the seven respondents will be deemed agreed and included in the final framework.

The criteria for inclusion in the framework was set at five of the seven respondents indicating they agreed or strongly agreed, which equates to 71 percent agreement. 

For each round of the Delphi, respondents were asked to indicate on the scale whether they agreed or disagreed with the inclusion of each stage in the framework, and then with each subsection of the stages. In addition to indicating their agreement/disagreement on the scale, there was a free text box to allow them to comment on their answer. The scores and comments from each respondent were reviewed and analysed. A document was compiled including every comment made by respondents. For each item, the mean and range of scores were noted. Following the first round, as no items were eliminated, this was solely for the information of the respondents and to allow them to consider what the group response was. In the second, third, and fourth rounds, participants were asked to go through the same process as in round one, indicating their level of agreement using the scale and to comment on their answer, while taking the feedback from the previous round into consideration. The mean scores, along with the range of scores (e.g., the marks ranged from three to five) as well as the anonymised comments from the preceding round were provided to respondents, but not the detail as to who made the comment or gave the score to the item.

There were several differences between the treatment of the data from rounds one and two. Following round two, any items rated as disagree or strongly disagree by at least five of the seven respondents were to be excluded, with all other items to be carried over to the third round. The other difference for this round was that the descriptions of items in the framework were modified based on the feedback in rounds one and two. Track changes was used for this to ensure that the changes made to the framework were clear, the same process was used for each subsequent round.

Following the third round, the same process took place as had occurred at the end of the second round, where the comments and scores were reviewed and the framework was modified based on the feedback. The difference between this round and the previous round was that the intention was for this to be the final round of the process. The level of consensus required for items to be included in the framework was that they needed to be rated as agree or strongly agree by at least five of the seven respondents. Those items were deemed to be agreed and included in the final framework. 

The next step in the study was a face-to-face meeting with all participants. Although this had not been planned at the outset, all members of the GFQACE were in attendance at the Life Long Learning in Pharmacy conference held in Brisbane, Australia in July 2018. The results of the study were presented to the GFQACE at a meeting in advance of the conference. Using the criteria as detailed above, two items did not meet the threshold required for inclusion and were therefore excluded from the final framework, and further detail on these items will be presented in the Results section. A discussion ensued, which demonstrated that a clear consensus had not been reached on the inclusion of these items. A face-to-face discussion does not typically form part of a conventional Delphi process, but it gave the participants a platform to air their views in more detail. It removed the degree of anonymity that typically characterises the process as all those involved freely expressed their opinions. Rather than attempting to reach consensus at the meeting, it was agreed by the group that both items would be modified in the framework based on feedback from the third round, in addition to the comments made at the meeting, and a further phase of the Delphi would be developed to exclusively cater for those two items. 

As ethics approval was granted for a three-round Delphi, it was necessary to apply for an amendment to the approval that was granted by the RCSI Research Ethics Committee. This included the circulation of a revised patient information leaflet (PIL) and consent form due to the introduction of the EU General Data Protection Regulation (GDPR). An email was sent to participants with the link to the form and an attachment that contained the framework. The same cut-off scores were used for this round as for the previous round. 

## 3. Results

The engagement with the process was excellent, with all seven participants representing the GFQACE organisations completing the questionnaire, and each of four rounds of the Delphi including the face-to-face meeting in Brisbane, a 100% response rate for all elements of the study.

Table 3 shows the mean scores for each stage and subsection across the four rounds of the Delphi. As can be seen in Table 3, the mean scores from respondents for the majority of items were between 4 and 5 for the Delphi, indicating agreement or strong agreement. Respondents were asked first to score and comment on the overall stages (e.g., input and process), followed by the subsections of these stages.

### 3.1. Delphi Round One

The results from round one showed that there was general agreement about the stages to be included in the framework, and the items to be included in each subsection. The item receiving the highest mean score overall was ‘application process’ at 4.9 out of a possible 5, and the lowest was ‘impact of activity’ at 3.7. As detailed in the cut-off scores, no items were removed from the framework following the first round.

Looking at some of the items in the framework, ‘evaluation of activity’, the item with the strongest levels of agreement, had comments that noted that it is “Essential for quality improvement” and “One of the only true measures”. The comments for ‘impact’, the lowest rated item, indicate much greater diversity of opinion: “Very important but perhaps sometimes challenging to measure objectively” and “How is the evaluation done?” Both of these comments potentially demonstrate that the issue with the inclusion of this item is linked to the practicalities of how it would be implemented, rather than with the concept and importance of impact.

### 3.2. Delphi Round Two

In round two, there was general agreement about the stages to be included in the framework, and the items to be included in each stage. The items receiving the highest mean scores in round two were ‘output’ and ‘accreditation standards’, both with 4.9. This was different to that of round one where the highest was ‘application process’. The lowest rated was again ‘impact of activity’ with a mean of 3.6. Two items received one score each of disagree: ‘method of delivery’ and ‘reflective practice’, and one item received one score of strongly disagree: ‘impact’. As a result of the scores, no items were removed from the framework following the second round. Some of the comments from rounds one and two indicated that they thought that an appeals process should form part of the framework “to dispute the outcome if they are not satisfied with the accreditation decision” and “to allow any provider to challenge the accreditation process and to ensure that their right to due process is safeguarded”. Based on this feedback, ‘appeals process’ was added to the output stage. Comments from respondents indicated that they appreciated having the opportunity to provide feedback, with many comments referencing those made in the first round, some agreeing with them, and others expanding on or clarifying them. This was interesting to see as it demonstrated the Delphi in action.

### 3.3. Delphi Round Three

The two items that received the highest mean score in round three were ‘application process’ and ‘output’, with both receiving a rating of strongly agree. The lowest rated was again ‘impact of activity’, with the mean staying consistent at 3.6. The criteria for inclusion or exclusion in the framework for the third round was that items rated as agree or strongly agree by at least five of the seven respondents were deemed agreed and included in the final framework. Two items did not reach the criteria for inclusion: ‘method of delivery’ and ‘impact’. These were the two lowest scoring items in round one, and two of the three lowest scoring items in round two. In round three, ‘method of delivery’ was rated as agree or strongly agree by four of the seven respondents, and ‘impact’ by three of the seven respondents. Only one respondent disagreed with the inclusion of ‘impact’ and their comment noted their concern on how to measure it.

### 3.4. Delphi Round Four

As detailed in the Methods section, this round was an additional round, following the face-to-face discussion at Brisbane. This round had just two items: ‘method of delivery’ and ‘impact’. Both items reached the level of consensus required to be included in the framework, which was that they needed to be rated as agree or strongly agree by at least five of the seven respondents. One respondent commented on why they thought some items might have received low scores initially:

For those items where ratings may have skewed towards neutral (rather than agree or strongly agree) with the previous Delphi, the issue was not a lack of agreement on the item being part of the quality framework, but rather some uncertainty on the stage the item was included or a desire for additional details on the description (i.e., “What is this?” and/or “Why is this required?”).

Details of the change to the wording for ‘method of delivery’ and ‘impact’ from rounds one to four of the Delphi can be viewed in Appendix B. As all items were deemed to be agreed upon following the fourth round, it appears that the modifications to the framework based on feedback from previous rounds, in addition to the discussion in Brisbane, were representative of the opinions of the majority of the respondents. Following round four of the Delphi, the overall structure was as shown in Table 2, with the addition of the ‘Appeals process’ subsection within the ‘Output’ stage. The final framework is included in Appendix C. Comments received on the framework were positive including “I like the revisions. Great job”, “It was excellent - so well laid out”, “I commend you on a well-designed, complete framework. You have captured the essential elements. It is easy to understand and follow” and “The framework captures the essential elements of quality CE.” One comment gave positive feedback on the framework and how it would fit in with the work being carried out by the GFQACE: “Great work. It is a great addition to our efforts on understanding CE accreditation and promoting mutual recognition.” These responses show that the framework achieved the aims set out at the beginning of the process.

## 4. Discussion

Accreditation has been widely written on, but often in terms of the accreditation of undergraduate training rather than that of CE activities. In 1996, the question was asked as to whether activities provided by accredited organisations resulted in better learning outcomes than those provided by unaccredited ones [29]. At that time, there was no clear answer. Twenty-four years later, there is still no clear answer on this. However, even if it is just that accreditation provides reassurance to the accrediting body and to the learner, this would seem a valid reason to have an accreditation process.

As anticipated, the information provided by participants in the questionnaire demonstrated that while there were many similarities across the organisations, there were also differences. Reasons for the differences can possibly be explained by the functions they fulfil, the culture and environment in which they are operating, and whether organisations were experienced or newly established. Based on the responses to the questionnaire, the inputs, process, and outputs from the accreditation processes across the GFQACE are fairly consistent, with occasional variations. This informed the development of the framework, where the commonalities informed the main structure and the variations identified areas where flexibility was required.

The proposed framework was developed following analysis of the results from the questionnaire and the documents submitted by the organisations, capturing the key themes and patterns that emerged from that phase of the study. The aim of the framework was to provide a guideline for accrediting an activity, considering all aspects of the accreditation process including the features of the activity itself, the governance and quality processes in place within the organisation seeking accreditation, the application and application review processes, the outputs from the accreditation process, and the ongoing process of quality review of the activity.

For both ‘impact’ and ‘method of delivery’, they each received scores of three points across the Delphi, something which is described as the central tendency, where respondents prefer to avoid the extreme ends of a scale, preferring instead to remain in a more neutral central position (which is variously referred to as “neither agree nor disagree”, or “undecided”), encouraging respondents to engage in satisficing, whereby they choose something that is “good enough” or acceptable [18,30,31,32,33,34,35]. This does not necessarily indicate disagreement with an item, but instead points to an element of uncertainty, possibly as to how it would be measured and/or implemented, and the comments bear that out. Only one respondent disagreed with the inclusion of ‘impact’ and their comment noted their concern on how to measure it. No respondents disagreed or strongly disagreed with including the ‘method of delivery’ in the framework.

As noted by other commentators, there is a law of diminishing returns with a Delphi. As the rounds progressed, the changes to the scores were often small, and by round three, there were substantially less comments. After round one, no changes were made to the framework based on the feedback, but all comments were viewed and noted. After round two, changes were made to the framework incorporating comments from rounds one and two. Not every comment was actioned as that would not be possible due to some being in opposition to others. Patterns were looked for in terms of what was appearing most frequently and there were a few themes that occurred in the responses. These included:

- The definition of quality assurance versus quality improvement.
- Transparency of the process.
- Clarification for items within the framework.
- Clarity for reviewers of applications.
- Consistency.
- Relevance.
- Importance of being realistic—where possible.
- Impact-how to measure.

At the meeting in Brisbane, most of the group agreed that ‘impact’ and ‘method of delivery’ were important items, and that further clarification on them would be of assistance. It was felt that with some small changes, ‘method of delivery’ could be clarified within the framework. The discussion mainly focussed on ‘impact’ as this seemed to be the most divisive issue. Comments in favour of its inclusion in the framework included:

- We should continue to strive for it.
- It should be a driver for change.
- We should do things that are difficult.
- Training providers should be held accountable to their mission statement.
- It should be modified so it is not beyond practice.
- It is part of quality improvement.
- It should be measurable.
- It is the difference between minimum standard, aspiration, and excellence.

Comments expressing concern included:

- We should recognise the limitations.
- Talking about change in society is beyond the scope of the accreditor.
- It is a factor beyond the remit of the provider.
- Learners do not think beyond their practice.

Overall the respondents recognised the importance of impact and how it allows the learner to determine if the activity is relevant for their practice, potentially leading to improved outcomes, but they also made reference to the practical difficulties of how impact will be measured and implemented, and to what the impact would refer to, whether that is the practice of the pharmacist, patient health, population health, or the practice of pharmacy.

With regard to ‘method of delivery’, comments noted that the method should be fit for purpose and relevant to the targeted participants, that it “must allow for different learner categories including learning styles and preferences, different practice backgrounds and work experience. Programme delivery must also be suitable for what is being taught.” One comment noted how the delivery has changed and queried the inclusion of facilities under method, as “Facilities often irrelevant, even in face-to-face. Increasing number of CE is web-based.” Others agreed that ‘method of delivery’ was an important subsection to include in the framework as “The breadth and changing nature of learning methods and the format for informing/teaching across these considerations suggest an obvious need to confirm the suitability of the method of delivery at the input stage.”

The acceptability of the proposed framework can be seen in the high levels of consensus on most items in the Delphi, and that the scoring was mainly in the “agree” and “strongly agree” end of the scale. The positive feedback from comments on the Delphi and at the face-to-face meeting of the GFQACE organisations in Brisbane demonstrated that participants were satisfied with the proposed framework. It was felt that the framework provided a useful starting point for the project on mutual recognition.

As discussed earlier, accreditation fulfils a number of roles, showing that an activity meets specified criteria and providing reassurance with regard to the quality of the activity. Accreditation has its origins in the education sector, with the first accreditation agencies appearing in the United States in the mid-1800s. The aim at that time was to standardise admission requirements, curricula, and duration of study in educational institutions, with the potential to allow students to transfer between institutions [36]. This can point towards the potential for using frameworks across disciplines given the importance of interprofessional practice, particularly in the healthcare area today.

There are a number of areas for which this research could be used in the future. It can provide guidance to organisations seeking to implement accreditation, or review processes currently in place. This work has demonstrated that despite the differences in the accreditation systems, that it is possible for organisations to reach consensus on a framework. The next, and perhaps more challenging step, would be to work on implementing the framework on an international level. This is perhaps related to the field of implementation science, which is based on real-world situations where findings from research are implemented into practice with the aim of improving quality and outcomes, and with a focus on patients, providers, organisations, and policies pertaining to healthcare [37,38]. It looks at potential gaps and “takes as part of its mission an explicit goal of developing generalizable knowledge that can be widely applied beyond the individual system under study” [38] (p. 3). This seems appropriate given that the work in this study looked at a group of organisations and produced something that has the potential to be applied beyond the boundaries of the original group. To develop this would require an implementation strategy, the development of relationships, and engagement with stakeholders to achieve buy-in [37,38]. This is certainly something to be considered for future work, outside of the remit of this research. Although the details of the framework refer to activities for pharmacists, the overall framework could be considered as somewhat professionally agnostic, and might therefore be suitable for use for the accreditation of activities for other professions.

With any study of this kind, it is important to acknowledge its strengths and limitations. The group used in the study was that of the GFQACE, which raises questions as to whether or not it is representative and can be applied to a larger population of other organisations that accredit CE activities for pharmacists. Although the organisations represented by the GFQACE are based in English-speaking countries, the participants were based in seven different countries in four continents, with their organisations fulfilling different functions, which brings more diversity to the results in the questionnaire and the opinions expressed in the Delphi. The sample that was used was the GFQACE, which was a group consisting of seven participants. The literature review showed that there is no ideal size for a sample and the expertise available in this group outweighs concerns over the size of the sample. Using a group of this size allowed for a more detailed analysis of the data from the questionnaire including the identification of commonalities than had a larger sample been used. By using the GFQACE as participants in the research, it meant that those participating had agreed that the work was required and that they were committed to involvement in the work, which was demonstrated in the 100% response rate.

## 5. Conclusions

The aim of the framework was to provide a potential roadmap for the accreditation of pharmacy continuing education activities and providers for organisations who may not have a procedure in place for this, or who may be looking to revise their systems. It provides a robust and quality-focussed model that takes into consideration all elements of accreditation to provide a framework for the accreditation of activities and draws on the procedures currently in place in the GFQACE, in the literature on the area, and on the FIP model.

Potential areas for future research could include expanding on reflective practice as this is still a developing area. It would be useful for more studies to be carried out on this, and for guidelines to be produced for organisations who are considering this as part of CE and CPD. Impact is something that needs further research, as has been shown by this study. In particular, guidance is required on how it will be measured and implemented in the context of CE.

It is planned by the GFQACE that the framework will now facilitate the work on mutual recognition. FIP has expressed interest in using the framework to inform its thinking in relation to accreditation of its activities. Should this progress, it will demonstrate a tangible output from this research.

## Figures and Tables

**Table 1:** The four sections of the questionnaire.

Section	Questions
Environment	Function(s) of organisation Types of activities accredited by organisation Name of the process for the recognition of CPD activities Details on governance structures for organisation
Application Process	Details on application process Documentation required from applicant Conditions to be eligible to apply for accreditation
Accreditation Process	Types of accreditation processes Aim of accreditation process Target learners for the programmes Details on standards or criteria Stages of the accreditation process Assessment of learning Any other approaches considered by the organisation
Output	Possible outcomes from the accreditation process Time limit on the duration of accreditation Circumstances in which the time period of accreditation can be changed Changes to the activity/provider Ongoing processes and/or requirements Changes to accreditation process

**Table 2:** Overview of accreditation framework as circulated in round one of the Delphi.

Input	Process	Output	Quality Improvement
Context for activity	Application process	Decision	Review of activity
Accreditation standards/principles	Application review process		Evaluation by participants
Quality processes			
Educational content			
Method of delivery			
Assessment approach			
Evaluation of activity			
Impact of activity			
Reflective Practice			

**Table 3:** Mean scores from each round of the Delphi.

Stage	Subsection	Round 1	Round 2	Round 3	Round 4
		Mean	Mean	Mean	Mean
Input	Overall	4.6	4.6	4.7	
Process	Overall	4.3	4.6	4.6	
Output	Overall	4.7	4.9	5	
QI	Overall	4.3	4.4	4.6	
Input	Context	4.3	4.7	4.9	
Input	Accreditation standards	4.4	4.9	4.9	
Input	Quality processes	4.6	4.4	4.3	
Input	Educational content	4.6	4.6	4.7	
Input	Method of delivery	3.9	3.9	4	4.4
Input	Assessment approach	4.6	4.6	4.7	
Input	Evaluation of activity	4.7	4.6	4.4	
Input	Impact of activity	3.7	3.6	3.6	4
Input	Reflective practice	4.1	3.9	4.1	
Process	Application process	4.9	4.7	5	
Process	Application review process	4.7	4.7	4.7	
Output	Decision	4.4	4.7	4.7	
Output	Appeals process			4.7	
QI	Review of activity	4.6	4.7	4.7	
QI	Evaluation by participants	4.4	4.6	4.3	

## Appendix A

Questionnaire circulated to the Global Forum on Quality Assurance of Continuing Education and Continuing Professional Development (GFQACE), prefaced by instructions and definitions.

### Accreditation Frameworks

This data collection tool (hereafter referred to as the questionnaire) has been sent to you as a representative of an organisation involved with the Lifelong Learning in Pharmacy Continuing Education Accreditation Steering Committee.

Before completing this questionnaire, you should have received a copy of the participant information leaflet and signed the consent form.

The questionnaire takes the following format:

- Questions relating to accreditation frameworks.
- Free text section at the end of the questionnaire which will allow you to input any information that you feel the questions have not covered.
- Supporting documentation—a list of documents is provided, and a link to upload them.

### Definitions

Different terms may be used for the process of accreditation of training programmes depending on the policy of an organisation. For clarity, the term ‘accreditation’ will be used throughout. These definitions are adapted from those in ‘Continuing Pharmaceutical Education: Guide to Establishing Quality Assured and Accredited Programs’, Accreditation Council for Pharmacy Education, 2016.

Accreditation: the process whereby recognition is granted to an organisation, site, or activity that meets certain established qualifications or standards.

Activity: An activity is an educational event designed to support the continuing professional development (CPD) of pharmacists to maintain and enhance their competence.

Continuing education (CE): a structured process of education designed or intended to support the continuing development of pharmacists to maintain and enhance their professional competence.

Continuing professional development (CPD): the process of active participation in lifelong learning activities that assists in developing and maintaining continuing competence, enhancing professional practice, and supporting achievement of career goals. A self-directed, ongoing, systematic, and outcomes focused approach to lifelong learning that is applied into practice.

### Environment

In this section, we are seeking information on the accrediting body.

- Name of person completing questionnaire.
- Name of your organisation.
- Describe the function(s) of your organisation, for example, regulatory body, provision of CPD activities, etc.
- Is your organisation involved in the accreditation of continuing education activities for pharmacy? If so, please provide a list of the types of activities accredited by your organisation.
- What is the name of the process for the recognition of CPD activities?
- Are there governance structures in place? If so, please describe.

### Application Process

In this section, we are seeking information on the application process.

- How does the applicant apply? (e.g., completion of hard copy paperwork, online application etc.)
- Please outline the documentation required to be submitted by the applicant.
- Are there any conditions the applicant must fulfil to be eligible to apply for accreditation?

### Accreditation Process

In this section, we are seeking information on the accreditation process.

- Are there different accreditation processes for different CPD activities (e.g., face-to-face/online training programmes, meetings, training materials, conferences etc.)?
- What is the aim of the accreditation process?
- Who are the target learners for the continuing pharmacy education programmes which are accredited by the organisation (e.g., pharmacists, pharmacy technicians, other healthcare professionals, a mixture of healthcare professionals)?
- Are there standards or criteria with which the applicant must comply in their application for accreditation?
- Describe all stages of the accreditation process in place in your organisation including application stage, assessment of the application, decision on accreditation, and timelines for these stages.
- Does assessment of learning typically form part of the training programmes accredited by your organisation?
- Have other approaches to accreditation been considered by your organisation? If yes, please provide details on this.

### Output

In this section, we are seeking information on the outputs from the accreditation process.

- Describe the possible outcomes from the accreditation process, for example, activity is accredited, activity is not accredited, activity is accredited on condition of fulfilling certain criteria, etc.
- Is there a time limit on the duration of accreditation of an activity/site/provider?
- Are there any circumstances in which the time period of accreditation can be changed?
- How are changes to the activity/provider managed?
- Are there ongoing processes and/or requirements that the provider must satisfy?
- In the course of this questionnaire you have described the accreditation process as it currently stands. If there have been changes made to the process, please outline them here.

### Additional Comments

Should you have any additional comments you would like to include on the accreditation framework within your organisation, please use this text box.

### Supporting Documentation

In addition to the questions above, we would appreciate it if the following documentation could be provided:

- Policy or guidance documents on accreditation if available.
- Legislative guidance (where relevant).
- Application form and/or any paperwork an applicant must submit relating to accreditation.
- Links to your organisation website etc. where data and documents can be found.
- Any other relevant information.

## Appendix B

**Table A1:** Wording for the subsection method of delivery, part of the input stage, showing the wording used in the first round and following the fourth round. The underlined text indicates where wording was added, the strikethrough text indicates where text was removed.

Rounds One and Two	What Is Required?	Why Is This Required?
	Delivery of an activity can take place in different formats-for example online, face-to-face, blended, etc. The applicant should furnish details on the following areas (where relevant): • Method of delivery • Expertise • Facilities	To ensure that the delivery meets the requirements of the learner.
After round four	**What is this**	**Why is this required?**
	This describes how the content will be delivered to the participants. Delivery of an activity can take place in different formats-for example online, face-to-face, blended etc. The applicant should furnish details on the following areas (where relevant): • Method of delivery • Expertise • Facilities • Virtual Learning EnvironmentThe applicant should indicate why this method of learning was chosen and why it is suitable for the activity and learners.	• To ensure that the delivery meets the requirements of the learner. Method of delivery should be relevant to the targeted participants, and the learning objectives and the content of the learning. • Method of delivery is important in the context of instructional design and use of teaching/learning methods to address educational needs and close practice gaps.

**Table A2:** Wording for subsection impact, part of the input stage, showing the wording used in the first round and following the fourth round. The underlined text indicates where wording was added, the strikethrough text indicates where text was removed.

Rounds One and Two	What Is Required?	Why Is This Required?
	This refers to the wider impact an activity can have on, for example, community health. A description from the applicant of what impact the activity is anticipated to have in practice.	Impact is an important element in measuring the success of an activity. It can be measured in different ways, and these can include impact on community health, patient health or practice of pharmacy as relevant.
After round four	**What is this**	**Why is this required?**
	This refers to the wider impact an activity can have on, for example, community health. the following areas: • individual practice • community population health • patient health • wider practice of pharmacy. Where possible, a description from the applicant of what impact the activity is anticipated to have should be provided.	• Impact is an important element in a quality framework and in measuring the success of an activity. It can be measured in different ways, and these can include impact on community health, patient health or practice of pharmacy as relevant. • CE can have a role in higher order outcomes/impact beyond satisfaction and knowledge acquisition. • Including expected impacts makes it possible for a prospective learners to take ownership of their learning and establish if the learning is likely to improve or expand their particular practice. Sometimes this provides insight to someone who ‘didn’t know what they didn’t know’. • Including ABCD (audience, behaviour, condition, and degree) of learning outcomes as an outcome statement would facilitate this.

## Appendix C

Final framework following completion of the Delphi process.

### Input

1. Context for Activity
  **What is this?**
  This is the background to the development of the activity. It could include, but is not limited to, the following items:
  - Needs assessment which could be by learners, and/or of learners, and taking into consideration the needs and the future directions of the profession.
  - Environment—the legislative basis and health profession requirements.
  - Changes in healthcare and pharmacy practice.
  - Alignment with stated local, national and professional needs and priorities.
  **Why is this required?**
  An activity is often the result of requests from pharmacists requiring training/learning in a new or expanded scope, which may be related to changes in funding mechanisms. It may also be a result of regulatory bodies identifying issues in the workplace that require addressing. Context ensures relevance of the activity by creating a relationship between the learning needs of the pharmacist and the environment within which they practise. Context enables pharmacists to see the relevance of CPD to their everyday practice and therefore to participate in effective learning. It ascertains that the activity is appropriate in the context of legislation, health service, and the direction of the profession.
  **Who provides this?**
  Applicant or accrediting body dependent on accrediting structures.
2. Accreditation standards/principles
  **What is this?**
  These are the standards or principles that have informed the development of the activity and to which the activity must adhere. In many cases, standards are provided by the regulator. Standards define measurable attributes that all CE activities must demonstrate to become accredited, and enables assurance that all accredited activities are consistent in terms of quality and relevance of learning. Standards can cover areas such as programme delivery, design, development, resources, evaluation, and governance.
  **Why is this required?**
  To ensure that the activity meets the required quality standards. These are objective minimums that the proposed activity must meet to be considered for accreditation.
  **Who provides this?**
  Accrediting body/regulator.
3. Quality Processes
  **What is this?**
  These are the processes that the applicant has put in place to assure content validity, quality of materials, and delivery. The applicant should evidence these processes by being required to include assurances that:
  - The activity design, content, assessment, and delivery are informed by and consistent with the stated learning objectives.
  - Anyone involved in activity development or presentation must be able to demonstrate they are suitably qualified/or experienced. Indicators of expertise may include (but are not limited to) the submission of key relevant experience including academic qualifications, credentials, and description of relevant current roles/responsibilities.
  - The activity is free of any commercial bias, and must not promote a particular product, service, perspective, or organisation.
  - Anyone involved in development (author, presenter, expert reviewers) must disclose any conflicts of interest whether actual or perceived to both the accrediting body and intending participants.
  - The processes for record keeping, feedback to participants, activity evaluation, and review are clear and well managed.
  **Why is this required?**
  To ensure that governance structures are in place. The content should be regularly reviewed and revised to reflect changes in best practice and pharmacy practice in general, and to ensure that sources are up-to-date and that content is unbiased.
  **Who provides this?**
  Applicant.
4. Educational Content
  **What is this?**
  This is the educational content of the activity. The content/materials should demonstrate how the activity intends to meet defined learning objectives created to address an identified educational need. A copy of the educational material will form part of the application and should demonstrate how the activity meets the objectives and outcomes
  **Why is this required?**
  Content for training programmes should be developed according to both objectives and outcomes. Examples of these can include:
  - Objectives that are observable and measurable (e.g., SMART (Specific, Measurable, Achievable, Realistic and Timely) or ABCD (Audience, Behaviour, Condition and Degree)).
  - Outcomes—knowledge, skills, behaviour.
  **Who provides this?**
  Applicant.
5. Method of Delivery
  **What is this?**
  This describes how the content will be delivered to the participants. Delivery of an activity can take place in different formats, for example, online, face-to-face, blended, etc. The applicant should furnish details on the following areas (where relevant):
  - Method of delivery
  - Expertise
  - Facilities
  - Virtual Learning Environment
  The applicant should indicate why this method of learning was chosen and why it is suitable for the activity and learners.
  **Why is this required?**
  Method of delivery should be relevant to the targeted participants, the learning objectives, and the content of the learning.
  **Who provides this?**
  Applicant.
6. Assessment Approach
  **What is this?**
  Assessment can be carried out by different methods, the most common of which are summative and formative assessment. The assessment approach should be detailed in the application.
  **Why is this required?**
  - Assessment assists with ensuring that participants are meeting the learning outcomes and provides a measurable benefit for them.
  - Assessment must be based on, and appropriate for, the stated learning objectives.
  - Feedback from assessment to be provided to learners where relevant and possible. It should be appropriate, timely, and constructive.
  - Assessment provides a metric for training providers on the performance of participants.
  **Who provides this?**
  Applicant.
7. Evaluation of activity
  **What is this?**
  Evaluation is an important aspect of the feedback on an activity. Provisions for how participant evaluations of the activity will be carried out should be detailed. Factors such as their learning experience (ease of achievement of learning objectives, relevance of activity to individual professional practice), overall satisfaction with the quality of the content, and relevance and effectiveness of delivery can be included in the evaluation.
  **Why is this required?**
  Programme improvement based on evaluations is an important aspect of good programme management by a provider and regular evaluation is important in the constantly evolving climate of healthcare. This ensures that a learner’s experience upon completion of the activity is captured and feedback is provided on the programme.
  **Who provides this?**
  Applicant or accrediting body dependent on accrediting structures.
8. Impact of activity
  **What is this?**
  This refers to the impact an activity can have on the following areas:
  - Individual practice
  - Population health
  - Patient health
  - Wider practice of pharmacy.
  Where possible, a description from the applicant of what impact the activity is anticipated to have should be provided.
  **Why is this required?**
  - Impact is an important element in a quality framework and in measuring the success of an activity.
  - CE can have a role in higher order outcomes/impact beyond satisfaction and knowledge acquisition.
  - Including expected impacts makes it possible for prospective learners to take ownership of their learning and establish if the learning is likely to improve or expand their particular practice. Sometimes this provides insight to someone who ‘didn’t know what they didn’t know’.
  - Including ABCD (audience, behaviour, condition, and degree) of learning outcomes as an outcome statement would facilitate this.
  **Who provides this?**
  Applicant.
9. Reflective Practice
  **What is this?**
  Reflective practice is a developing area in CPD, which allows the participant to consider how they can integrate their learning into their practice. Providers of continuing education have a role in serving as a partner in professional development and helping learners develop self-directed lifelong learning skills.
  The applicant should be encouraged to provide for the participant’s engagement in reflection in the context of their own practice. This could, for example, take the form of completion of a CPD cycle, or asking the participant to consider questions such as How can I apply this in practice? What do I intend to do differently? etc.
  **Why is this required?**
  Where possible, activities undertaken by pharmacists as part of their continuing education should take into consideration the following areas:
  - Practice and behavioural changes
  - Personal development
  - Relevance
  - Applicability
  **Who provides this?**
  Applicant

### Process

1. Application Process
  **What is this?**
  This refers to the application process. An application form and guidance should be provided to the applicant. The applicant should provide the following items as part of the process:
  - Application form, mapped to inputs.
  - Application fee, where relevant.
  - Declaration of conflicts of interest.
  **Why is this required?**
  The application documentation is required to demonstrate how the activity meets the standards, outcomes, and objectives. An activity must demonstrate that it satisfies all of the accreditation requirements to be accredited. To ensure that the content of the programme is unbiased, presenters and authors must declare any conflicts of interest, all third parties must be clearly acknowledged in the programme, and all conflicts highlighted to participants.
  **Who provides this?**
  Applicant.
2. Application review process
  **What is this?**
  This is the process of reviewing the application submitted by the applicant. The role of the reviewer, their qualifications, experience, and suitability should be clearly defined. Reviewers selected should meet the following criteria:
  - Have the appropriate expertise to review the application.
  - Have an understanding of content validity, educational and assessment processes.
  - No conflicts of interest.
  The following are the steps in the review process:
  - Activity to be reviewed.
  - Review team to issue recommendation.
  **Why is this required?**
  To ensure that the standards have been met, that the application is complete, and that the activity is fit for purpose. The programme needs to be reviewed for current relevance, accuracy of content, and author/presenter credentials.
  **Who provides this?**
  Accrediting body

### Accreditation Output

1. Decision
  **What is this?**
  (a) Accreditation granted.
  (b) Accreditation not granted.
  (c) Accreditation granted once the programme has met conditions as detailed in the report.
  **Why is this required?**
  This is the output from the application review process. The review team decision/recommendation is to be determined and communicated to the applicant. This should include any conditions or recommendations by the review team as well as the duration of the accreditation.
  **Who provides this?**
  Accrediting body.
2. Appeals Process
  **What is this?**
  An appeals process allows the provider to appeal the output from the review process.
  **Why is this required?**
  An appeals process is required to ensure the transparency of the process, and that the right to due process is safeguarded.
  **Who provides this?**
  Accrediting body to facilitate.

### Quality Improvement

1. Review of activity
  **What is this?**
  QI is a continuous improvement process to review, critique, and implement positive change. It is a proactive approach to better systems leading to improved outcomes. The activity should be reviewed on a regular basis, taking the following into consideration (where relevant and possible):
  - Legislation
  - Current clinical guidance
  - Software updates
  - Evaluations by participants
  - New research evidence
  - Mean scores on learning assessment
  - Skill-based exams
  - Observation in practice/simulation
  **Why is this required?**
  Regular review of activities by the applicant ensures that they continue to meet the learning outcomes and objectives, while confirming that the content is updated to adhere to best practice.
  The accrediting body has an ongoing responsibility to their programme users to ensure that the quality of any accredited programme is maintained and improved.
  **Who provides this?**
  Applicant.
2. Evaluation by participants
  **What is this?**
  Evaluation is an important aspect of the feedback on an activity. Provisions for how participant evaluations of the activity will be carried out should be detailed.
  **Why is this required?**
  Evaluation of an activity by participants assures that it is regularly reviewed for quality purposes. This allows the applicant to receive feedback on the experience participants have while undertaking the activity. This must assess their view of:
  - Their achievement against the stated learning objectives.
  - The relevance of activity and content to their practice.
  - Their overall satisfaction with the activity as a whole.
  - The suitability of delivery of the activity.
  **Who provides this?**
  Applicant or accrediting body dependent on accrediting structures.

## References

[1] Rosof A., Felch W.C. (2004). Continuing Medical Education: A Primer.

[2] Rouse M.J. (2004). Continuing professional development in pharmacy. Am. J. Health Syst. Pharm..

[3] Austin Z., Marini A., Glover N.M., Croteau D. (2005). Continuous professional development: A qualitative study of pharmacists’ attitudes, behaviors, and preferences in Ontario, Canada. Am. J. Pharm. Educ..

[4] Austin Z. (2013). CPD and revalidation: Our future is happening now. Res. Soc. Adm. Pharm..

[5] Eva K.W., Bordage G., Campbell C., Galbraith R., Ginsburg S., Holmboe E., Regehr G. (2016). Towards a program of assessment for health professionals: From training into practice. Adv. Health Sci. Educ..

[6] Stevenson R., Moore D.E. (2018). Ascent to the summit of the CME pyramid. JAMA J. Am. Med. Assoc..

[7] Osler W. (1900). An Address on the Importance of Post-Graduate Study. BMJ.

[8] (2016). International Academy for CPD Accreditation Glossary. https://academy4cpdaccreditation.files.wordpress.com/2016/11/iacpda-glossary-final-2016.pdf.

[9] De Andrade F., Griebenow R., Costello R.W., Guenova M., Schaefer R., Chalmers J.D., Tichelli A., Raguz D., Stein J. (2018). The future of accreditation of continuing medical education (CME)—Continuing professional development (CPD) in Europe: Harmonisation through dialogue and consensus. J. Eur. CME.

[10] Martin M., Hernes G., Martin M. (2008). Policy rationales and organizational methodological options in accreditation: Findings from an IIEP research project. Accreditation and the Global Higher Education Market.

[11] (2010). Review of International CPD Models.

[12] Greenberg M. (2014). It’s Time for a New Definition of Accreditation. The Chronicle for Higher Education. https://www.chronicle.com/article/Its-Time-for-a-New-Definition/144207.

[13] Accreditation Schemes.

[14] Phillips S.D., Kinser K. (2018). Accreditation on the Edge: Challenging Quality Assurance in Higher Education.

[15] Eaton J.S. (2015). An Overview of U.S. Accreditation.

[16] Braithwaite J., Westbrook J.I., Johnston B., Clark S., Brandon M., Banks M., Hughes C.F., Greenfield D., Pawsey M., Corbett A. (2011). Strengthening organizational performance through accreditation research—A framework for twelve interrelated studies: The accredit project study protocol. BMC Res. Notes.

[17] Stone D.H. (1993). How to do it: Design a questionnaire. BMJ.

[18] Krosnick J.A., Presser S., Marsden P.V., Wright J.D. (2010). Question and questionnaire design. Handbook of Survey Research.

[19] Bryman A. (2006). Integrating quantitative and qualitative research: How is it done?. Qual. Res..

[20] Östlund U., Kidd L., Wengström Y., Rowa-Dewar N. (2011). Combining qualitative and quantitative research within mixed method research designs: A methodological review. Int. J. Nurs. Stud..

[21] Paltved C., Musaeus P. (2012). Qualitative research on emergency medicine physicians: A literature review. Int. J. Clin. Med..

[22] Wisdom J., Creswell J.W. (2013). Mixed Methods: Integrating Quantitative and Qualitative Data Collection and Analysis While Studying Patient-Centered Medical Home Models.

[23] Creswell J.W. (2014). Research Design: Qualitative, Quantitative, and Mixed Methods Approaches.

[24] Palinkas L.A., Horwitz S.M., Green C., Wisdom J., Duan N., Hoagwood K. (2015). Purposeful sampling for qualitative data collection and analysis in mixed method implementation research. Adm. Policy Ment. Health Ment. Health Serv. Res..

[25] Almalki S. (2016). Integrating quantitative and qualitative data in mixed methods research—challenges and benefits. J. Educ. Learn..

[26] (2014). Quality Assurance of Pharmacy Education: The FIP Global Framework.

[27] Meštrović A., Rouse M.J. (2015). Pillars and foundations of quality for continuing education in pharmacy. Am. J. Pharm. Educ..

[28] Rouse M.J., Vlasses P.H., Wadelin J.W., Zarembski D.G., Joshi M.P., Mabirizi D., Saleeb S.A. (2016). Continuing Pharmaceutical Education: Guide to Establishing Quality Assured and Accredited Programs.

[29] Mazmanian P.E., Mazmanian P.M., Moore M.E. (1996). Evaluation-based accreditation: Alternative approach to regulating continuing medical education providers. J. Contin. Educ. Health Prof..

[30] Johns R. (2010). SQB Methods Fact Sheet 1: Likert Items and Scales.

[31] Peng S. (2013). Maximizing and satisficing in decision-making dyads. Whart. Res. Sch..

[32] Rinker T. (2014). On the Treatment of Likert Data. https://www.researchgate.net/publication/262011454_Likert.

[33] Bishop P.A., Herron R.L. (2015). Use and misuse of the Likert item responses and other ordinal measures. Int. J. Exerc. Sci..

[34] Trevelyan E.G., Robinson P.N. (2015). Delphi methodology in health research: How to do it?. Eur. J. Integr. Med..

[35] Douven I. (2018). A Bayesian perspective on Likert scales and central tendency. Psychon. Bull. Rev..

[36] Flores A. (2015). Hooked on Accreditation: A Historical Perspective.

[37] Bauer M.S., Damschroder L., Hagedorn H., Smith J., Kilbourne A.M. (2015). An introduction to implementation science for the non-specialist. BMC Psychol..

[38] Handley M.A., Gorukanti A., Cattamanchi A. (2016). Strategies for implementing implementation science: A methodological overview. Emerg. Med. J..

